# Electro-Driven Materials and Processes for Lithium Recovery—A Review

**DOI:** 10.3390/membranes12030343

**Published:** 2022-03-18

**Authors:** Anna Siekierka, Marek Bryjak, Amir Razmjou, Wojciech Kujawski, Aleksandar N. Nikoloski, Ludovic F. Dumée

**Affiliations:** 1 Department of Process Engineering and Technology of Polymeric and Carbon Materials, Wroclaw University of Science and Technology, Wybrzeże Wyspiańskiego 27, 50-370 Wrocław, Poland; marek.bryjak@pwr.edu.pl; 2 School of Engineering, Edith Cowan University, 270 Joondalup Drive, Joondalup, Perth, WA 6027, Australia; amir.razmjouchaharmahali@uts.edu.au; 3 Centre for Technology in Water and Wastewater, University of Technology Sydney, Sydney, NSW 2007, Australia; 4 Faculty of Chemistry, Nicolaus Copernicus University in Toruń, 7 Gagarina Street, 87-100 Toruń, Poland; 5 College of Science, Health, Engineering and Education, Murdoch University, Perth, WA 6150, Australia; a.nikoloski@murdoch.edu.au; 6 Department of Chemical Engineering, Khalifa University, Abu Dhabi P.O. Box 127788, United Arab Emirates; ludovic.dumee@ku.ac.ae; 7 Centre for Membrane and Advanced Water Technology, Khalifa University, Abu Dhabi P.O. Box 127788, United Arab Emirates; 8 Research Center on CO2 and Hydrogen (RICH), Khalifa University, Abu Dhabi P.O. Box 127788, United Arab Emirates

**Keywords:** lithium compounds, electro-membrane processes, brines, minerals, e-waste

## Abstract

The mass production of lithium-ion batteries and lithium-rich e-products that are required for electric vehicles, energy storage devices, and cloud-connected electronics is driving an unprecedented demand for lithium resources. Current lithium production technologies, in which extraction and purification are typically achieved by hydrometallurgical routes, possess strong environmental impact but are also energy-intensive and require extensive operational capabilities. The emergence of selective membrane materials and associated electro-processes offers an avenue to reduce these energy and cost penalties and create more sustainable lithium production approaches. In this review, lithium recovery technologies are discussed considering the origin of the lithium, which can be primary sources such as minerals and brines or e-waste sources generated from recycling of batteries and other e-products. The relevance of electro-membrane processes for selective lithium recovery is discussed as well as the potential and shortfalls of current electro-membrane methods.

## 1. Introduction

Lithium (Li) metal is a unique element exhibiting the most negative redox potential, equal to −3.014 V compared to a standard hydrogen electrode, making it extraordinarily reactive and valuable across multiple electrochemical applications [1]. Li is also the lightest non-iron metal, with a density of 0.53 g/cm^3^, and has the third-highest specific heat capacity (Cp = 3.6 J g^−1^ K^−1^). These exceptional properties have supported the emergence of Li-based devices in applications where higher power densities and the long-lasting life of energy storage devices are required [2].

**Figure 1:**
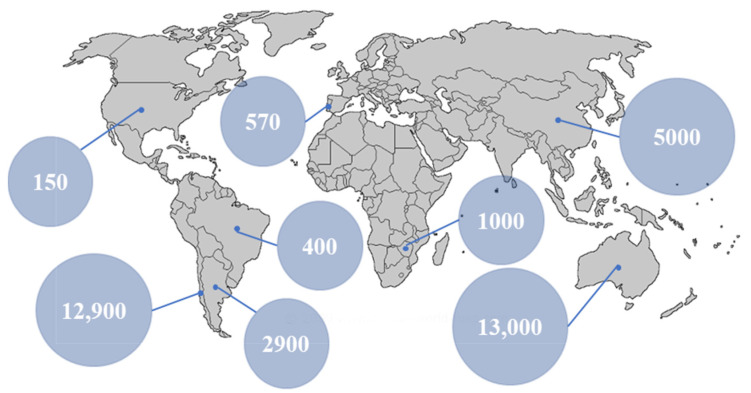
World production of lithium in 2017 calculated in ton/year. Reprinted based on the open access license [11].

Lithium resources can be divided into either primary or secondary sources. Primary sources include mineral rocks [3], saline lakes [4], brines [5], sea water [6], underground water [7], and groundwater [8], while secondary sources are typically lithium obtained from recycling lithium-containing devices and components, such as batteries, capacitors [9], or general e-wastes [10]. The world’s primary reserves of lithium are estimated at over 250 billion tons [1], of which 230 billion tons are present within oceans, while the remaining amount exists as ores or continental brines. The demand for Li metal has increased exponentially over the past 20 years and was 33,300 tons in 2015. The global production of lithium in 2017 [3] primarily originated from Australia, China, Argentina, and Chile (Figure 1).

Driven by current demands for lithium and limited resources, spot lithium carbonate prices in China have increased by 300% and, recently, briefly exceeded USD 20,000 per ton due to an acute but temporary shortage of imported spodumene from Australia. However, this is an indicator of future long-term market sensitivity and trends. Although Li compounds are used in glass and ceramics manufacturing (30%), as a component during lubricants synthesis (8%), and in air purification (3%) and polymer production (5%), its largest application is in batteries and capacitors (39%) [5]. This use will increase exponentially in future years due to sustainability-focused policies and the increasing dominance of electric vehicles [12]. The consumption of lithium will also be stimulated by emerging applications for Li such as rocket fuel, where high energy densities and specific impulses are required for take-off, and in Li-based alloys and lithium hydride production [13,14]. Based on the current price at USD 100,000 per ton, the market for Li-metal would reach USD 500 billion by 2050 [15].

Current commercial lithium production strategies from primary sources are primarily based on continental (59%) and geothermal brines (3%), as well as ores in the form of hard rock (25%) and hectorite (7%) minerals. Over 130 minerals containing lithium have been identified and exploited at industrial scales, including silicates and phosphate-based ores [16,17]. Although spodumene, pegmatites, petalites, lepidolite, amblygonite, zinnwaldite, and eucryptite offer theoretical lithium contents between 3 and 5.53% [18], the achieved concentrations rarely exceed 0.5 to 2% (Table 1) [5,18].

Lithium may also be extracted from surface and ocean waters (Table 2). Although ocean waters contain between 0.1 and 0.2 ppm Li, there is no cost-effective technology available now to extract Li at such low levels. On the order hand, the Li concentration of 10 to 20 ppm within geothermal brines is much more attractive, but challenges related to the presence of other highly concentrated metals ions, such as arsenic, mercury, or boron, render selective extraction challenging [19,20]. Salt lake brines are amongst the most concentrated naturally occurring sources of Li ions, ranging from a few hundred to thousands of ppm. However, a critical challenge in extracting lithium from this source also relates to the presence of interfering ions which contribute to water hardness, such as calcium and magnesium. The ratio of Mg^2+^/Li^+^ is typically larger than 40 and can be as high as 200 in some extreme cases.

Secondary sources of lithium arise from the recycling of e-waste materials, including batteries and capacitors. The amount of Li across such electronic parts dramatically varies based on brands and fabrication technologies, but these materials still typically represent a significant component. There is also an imperative to re-use spent Li-containing components rather than sending environmentally damaging material to waste given the current global focus on the Circular Economy. In 2015, at least 5600 million LIB cells were sold worldwide, and the LIB market size is forecasted to increase by another 10.6% from 2016 to 2024, reaching a market value of USD 56 billion by 2024 [21]. LIBs contain 2 to 7 wt% of Li, a concentration significantly higher than that present in natural ores or within marine sources, making extraction from spent batteries attractive as an essential secondary source of Li (Table 3).

Conventional LIB materials include LiCoO_2_ [23], LiMn_2_O_2_ [24], LiNi_0.33_Mn_0.33_Co_0.33_O_2_, or LiFePO_4_ [25]. One ton of spent LIBs cathode battery waste represents approximately USD 8500 of Li and USD 7200 of Co [9]. The LIBs electrolyte, whose role is to support the rapid transportation of carrier ions across the electrodes, is typically composed of LiPF_6_ [26] with additives such as NaPF_6_ [27] or LiBF_4_ [28].

The previous review published in 2021 dealt with lithium recovery through green electrochemical-battery approaches [29]. The authors focused on challenges for lithium extraction from battery wastes by the application of an electrochemical battery system employed with a lithium-capturing electrode for Li recovery [30]. Another review, which was also published in 2021, dealt with challenges for lithium supply, focusing on the life cycle of lithium and its recovery following circular economy rules [29]. Moreover, Kader et al. summarized the techniques of lithium recycling from lithium-ion batteries [31]

From these reviews, it is clear that challenges in efficient and cost-effective separation are still limiting the cost-effectiveness of Li production, and advances in techniques for the selective speciation of Li from complex brines and effluents are required. This review discusses the potentials of electro-membrane processes to support mining and hydrometallurgical operations, as well as the recycling and recovery of Li from used items and devices. The application of electro-membrane processes supporting the speciation of Li will be presented and critically discussed in terms of ion selectivity, Li recovery efficiency, the theory of specific capturing Li, and techno-economical aspects. A circular Li economy will only arise from the synergistic development of intensive and integrated technologies trains. The prospects for electro-membrane processes to contribute to this technology paradigm will be discussed.

## 2. Benchmark Lithium Compounds Production Technologies

The current methods of producing lithium compounds vary with the origin of the feedstock, whereby Li-ions, as well as other valuable metal ions, are extracted. In the following sections, technologies are therefore divided into methods applicable to mineral rocks, brines, and lixiviate from e-waste digestion. This section will briefly benchmark existing commercial technologies to enable subsequent comparisons with electro-membrane processes.

### 2.1. Conventional Recovery of Li from Ores

In extractive metallurgy, Li is recovered chemically or through a combination of chemical and pyro-metallurgical processes. Two different processes, namely, roasting and calcination or chlorination and leaching, are reported to support Li recovery from ores. These processes involve calcination or roasting followed by leaching to dissolve lithium and transfer it into an aqueous phase. Following typical ore processing techniques, such as grinding, filtration of slurries, and water recovery processes, Li may be selectively produced and extracted from mineral ores by leaching processes either in acidic or alkaline aqueous solutions [5,11,17,18]. The first stage of the chemical processing on hard rock Li mineral-bearing ores these days typically involves the sulfuric acid pug roasting of the mineral ore at a temperature between 250 and 400 °C to support the decomposition of the silica mesostructure and convert the Li contained in the minerals into a water-soluble form [32]. Alkaline processes, whereby minerals are reacted with a mixture of calcium sulfate and calcium oxide/hydroxide at ~250 °C to convert the silicate into water soluble Li aluminate can also be performed to yield LiOH or Li_2_CO_3_ salts [33]. Ion exchange processes are sometimes required to support the extraction of undesired components and increase the purity of the Li product liquor [6,34,35].

During acid/sulfonation processes, alkali metal sulfates, sulfuric acid, or SO_3_ gas mixed with water and oxygen are employed as reagents to produce highly water-soluble Li sulfates that are less prone to precipitation compared to other Li compounds. However, drawbacks include the large volumes of reagent chemicals required and challenges in producing high-purity Li carbonates from such brines resulting from the capacity of sulfate reagents to bind to Al, Na, Mg, Fe, and K [2,36]. The sulfate roasting of lepidolite followed by water leaching has been studied widely using Na_2_SO_4_/K_2_SO_4_/CaO, Na_2_SO_4_, and Fe_S_O_4_ and has yielded Li extraction extents up to 99.5% at 1000 °C [37,38,39]. The use of Na_2_SO_4_ and H_2_SO_4_ with the zinnwaldite, petalite, and montmorillonite ores has yielded Li extraction extents up to 90, 97.3, and 90%, respectively [5,37,40,41]. However, this approach normally requires sodium carbonate dosing to precipitate Li carbonates [5].

The alkaline Li extraction process is a more economical and more environmentally benign process that involves Li extraction from minerals with lime as an active leaching reagent. The roasting of Li ores in the temperature ranges 100–205 °C and 825–1050 °C will convert Li ores to LiO_2_, a precursor to LiOH. The lithium hydroxide produced can be further converted to LiCl or LiCO_3_ by a reaction with hydrochloric acid or carbon dioxide [5]. The lithium precipitates may be further upgraded while the mother liquor, such as the liquor obtained after lithium crystallization, is looped to the first stage of the process.

The chlorination of lithium concentrates takes place between 800 and 1100 °C in the presence of hydrochloric acid, sodium chloride, calcium chloride, or chlorine gas, depending on the original ore chemistry. The process is used to convert the lithium compounds into lithium chloride (LiCl), which can be solubilized in water and thus purified. As an example, an acid baking process involving roasting of β-spodumene with Cl_2_ gas at 1100 °C for 2.5 h resulted in almost complete extraction of Li as LiCl_2_ [42]. The systems utilized for lithium recovery from minerals are summarized in Table 4.

### 2.2. Conventional Recovery of Li from Brines

The comparison the different type of conventional method of lithium extraction from brines are summarized in the Table 5.

Brines have become one of the most popular sources of Li ions since Li extraction requires fewer pre-treatments than from ores and a large variety of Li salts are available, as well as the relatively high concentration of Li in brines, as well as from the ratio of rare earths and alkaline metals to lithium ions, supporting the co-regeneration of various valuable compounds. Brines may be divided into three types, including brines generated during the evaporation processes, directly extracted from geothermal and underground sources, and aqueous liquors produced from oil/petroleum fields [6]. The traditional methods of production of Li compounds from brines include evaporation, column adsorption, and diffusion dialysis, which leads to Li-enriched solutions that are further augmented in Li by ion exchange, sequential adsorption, or solvent extraction [34].

Ion exchange (IEX) resins are amongst the most used technologies to extract Li from brines. Commercial IEX materials including MC50 (Chemie AG, Bitterfeld-Wolfen, Germany), TP207 (Bayer AG), and Y80-N Chemie AG (Chemie AG, Bitterfeld-Wolfen, Germany) have been used for the separation of Li from synthetic brines [44]. Li extraction from the Dead Sea waters using ionic liquids such as triisobutyl phosphate [45] and liquid chromatography using polyactylamide Bio-Gel P-2 and Blue Dextran 2000 were also demonstrated [6], supporting selective extraction against Mg^2+^ and Ca^2+^ ions. Hybrid ion exchangers based on inorganic adsorbents or aluminate salts were also tried to effectively capture Li ions from brines. The inorganic ion exchanger H_2_TiO_3_ was used to separate lithium from the Uyuani lake in Bolivia, where the Li-ion adsorption capacity was estimated at 32.6 mg/g (4.8 mmol/g) at a pH of 6.5 [46]. It was possible to apply the cation exchanger titanium (IV) antimonate to reduce the content of K^+^, Mg^2+^, and Ca^2+^.

An attractive set of technologies to generate Li cost-effectively from aqueous solutions involve membrane processes, including pressure-driven processes. Reverse osmosis (RO) and nanofiltration (NF) have been used to concentrate and separate lithium ions selectively [47]. NF90 membranes yielded 85 wt% separation of Li^+^ from Mg^2+^ salts with a relatively low desalination range of about 15 wt% of lithium. These membrane processes may be intensified towards the speciation of mixed Li and boron from geothermal water by combining membrane technologies with adsorbents [20]. Dowex XUS-43594 combined with λ-MnO_2_ ion exchange resins supported the selective extraction of Li and boron at 100% and 83%, respectively [20]. Membrane distillation coupled to crystallization processes has also been considered for Li recovery. Direct contact membrane distillation and osmotic membrane distillation processes achieved a degree of saturation of LiCl in an aqueous solution. Electro-membrane processes based on electrodialysis and capacitive deionization have been developed and demonstrated and will be discussed in more detail in Section 3.

**Table 5:** Comparison of processes for lithium extraction from brines [48,49,50,51,52].

Process	Adsorption	Membrane-Type Technologies	Thermal Technologies
Active reagents	Ion exchange resins, sorbents such as activated carbon or spinel-type materials	Ion exchange membranes, porous and nonporous membranes, asymmetrical with active thin layer	Thermal energy from sun light
Time	12–24 h	12–24 h	>45 days
Temperature	25 °C	25 °C	Depends on the region of evaporation (25–35 °C)
Disadvantages	Sorption and desorption operation are required; batch operation; column package consumes a lot of resin (>0.5 kg); pretreatment is required	Fouling of membranes; stack of member to be effective; costs of membranes; required the separation and concentration nexus; pretreatment is required; required driven forces	Long-lasting process; small amount of brine rich in Li^+^ salts; low selective method
Advantages	Flexibility of application depends on the type of resin; high selective; long-lasting time of using	High selectivity; continuous operations; flexibility of application	High concentrations of Li salts are obtained

### 2.3. Conventional Li Recovery from e-Waste Products and Process Liquors

Li-ion extraction from recycling of e-waste materials can be achieved through hydrometallurgical and pyro-metallurgical methods [21]. Mechanical pre-processing is required to generate individual streams of Li-rich waste.

Pyro-metallurgical methods involve high-temperature operations, where redox reactions are activated to smelt and purify valuable metals. Pyro-metallurgical methods are typically combined with hydrometallurgical methods, which involve the leaching of valuable elements from a solid matrix and their subsequent precipitation by solvent-phase separation [21].

Pyro-metallurgical processes are performed at a temperature range between 800 and 1000 °C [53]. LiCoO_2_ with commercially required properties was generated by pyro-metallurgical processing of crushed LIBs calcined in air at 850–950 °C for 12 h [54]. Oxygen-free roasting combined with wet magnetic separation and the regeneration of cobalt and lithium carbonates was also performed at 1000 °C, resulting in the recovery of 95.72% and 98.93%of Co and Li_2_CO_3_, respectively [55]. Vacuum metallurgy was also used for LIB waste processing, and both Li_2_CO_3_ and Mn_3_O_4_ were obtained by heating at 800 °C under vacuum conditions, yielding purities of 91.3% and 95.1%, respectively [56].

Hydrometallurgical processes involve the extraction by leaching valuable metals from the LIBs and the subsequent recovery of the dissolved metal ions, including Li from the generated liquors. This is a mature technology, and a number of optimization studies of the leaching conditions such as reagent type and dosage, leaching rate and duration time, pulp density, and temperature have been performed. Such leaching processes may be performed in various alkali or acid leaching systems under different redox conditions. Alkali leaching is typically more selective and reduces the number of purification steps required. For example, ammonia-based systems are utilized since ammonia may form stable and selective complexes with transition metal ions [57,58]. Different behavior is exhibited by manganese, where the success of the complexation reaction is strongly related to the concentration of the ammonia agent [58]. The acidic extraction systems from LIB wastes remain prevalent compared to alkaline ones, as they often offer high recovery efficiencies. However, the use of strong inorganic acids may lead to product contamination, which is difficult to remediate. The most efficient inorganic acids leaching agents are HCl [59], H_2_SO_4_ [60], and HNO_3_ [61], while organic acid leaching agents include citric [62], ascorbic, oxalic [63], and formic acids [64]. The choice of leaching agent has a strong influence on economic aspects of the process, as well as on environmental aspects and the production and/or reduction of by-products [61]. However, typically, hydrometallurgical technology is characterized by high recovery efficacy, low energy requirements, and high reaction rates.

Biometallurgy or bioleaching is a recently developed technology for the extraction of valuable metals from spent LIBs, whereby microbial metabolism or microbial acid production processes are used to extract the metals from effluents. Bacteria digestion will generate inorganic acid, while fungi digestion may form organic acids. A key drawback of bioleaching is the long culturing time and the susceptibility of the biological agents to contamination and poisoning. An indirect, non-contact bio-hydrometallurgy process for polymetallic waste processing was proposed, whereby biological reagents, produced by *Acidithiobacillus ferrooxidans* DSM 14882T and *Acidithiobacillus thiooxidans* DSM 14887T, were mixed with 100 mM H_2_SO_4_ into a biogenic ferric solution to achieve leaching yields of 53.2% for Co, 60.0% for Li, 48.7% for Ni, 81.8% for Mn, and 74.4% for Cu [65]. The traditional techniques of LIB leaching by sulfuric acid applied the 2 M of H_2_SO_4_ (T = 80 °C, t = 60 min). The following recovery efficiencies could be attained: 98.7% for Ni, 97.1% for Mn, 98.2% for Co, and 81.0% for Li under optimized experimental conditions [66]. The biometallurgy method compared with the traditional method obtain a lower efficiency of extraction metals from LIBs. Considering the 20 times lower concentration of extractant, the results with biological reagents are promising.

The metal recoveries from the use of pyro-metallurgy, hydrometallurgy, and bio-metallurgy for spent LIBs recycling are shown in Table 6.

## 3. Electro-Membrane Processes for Lithium Recovery

Electro-membrane processes, including electrodialysis (ED) and capacitive deionization (CDI), are described and discussed in terms of Li recovery and extraction efficiencies in this section. The relationships between the source materials’ intrinsic properties and their response to electrical current and voltage applications are also presented.

### 3.1. Lithium Extraction from Brines by Electrodialysis

Electrodialysis (ED) is a mature membrane-based separation process, developed in the 1950s, allowing for the specific ions’ speciation across ion-exchange membranes. ED was primarily applied and scaled up for the purification of industrial wastewaters, fine chemical broth deionization, as well as ultra-pure water production. The permeation of cations and anions across the respective cation and anion exchange membranes is achieved upon application of an electrical potential difference [68,69,70].

#### 3.1.1. Principle of Electrodialysis and Materials Considerations

**Figure 2:**
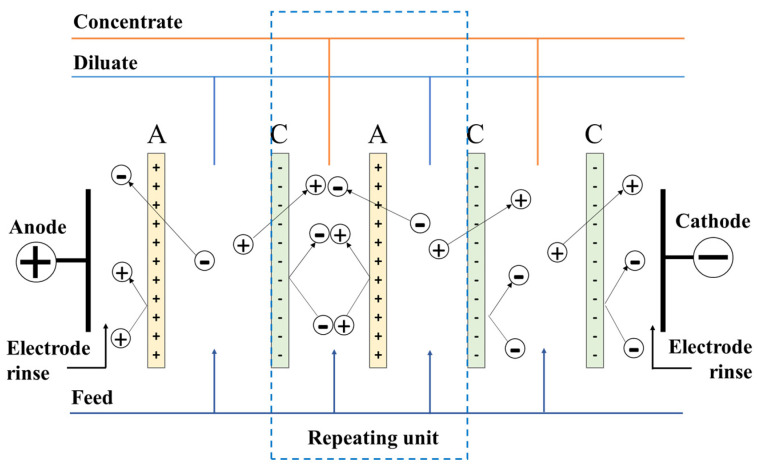
Schematic diagram of principles of classic electrodialysis.

The ED membranes may be designed from a single type of ion exchange material to create charge- or valence-selective membranes, or they can be assembled in layers of alternating cation and anion exchange components, leading to bipolar membrane systems, used extensively in fuel cells for selective proton transfers [71]. In a typical process, flat-sheet membranes are assembled in stacks between two electrodes that are used to generate a potential difference leading to ion diffusion within the diluate and membrane materials. The general operation of electrodialysis is illustrated in Figure 2.

The process relies on the effect that in high-saline aqueous systems, the mass transfer is significantly affected by the complexity of ions, where the main role in the transfer is due to the steric hindrance and charge effect. The hydration radius of monovalent cations is smaller than divalent cations, which leads to the ability to attract free water molecules to the ionic center [8]. Furthermore, considering the hydration potential, which indicates how strongly an ion would its lose water shell, the influence sequence of coexisting cations was explained legitimately. The decline in water shell envelope cations is strongly dependent on the concentration and existing co-ions in aqueous solution.

**Figure 3:**
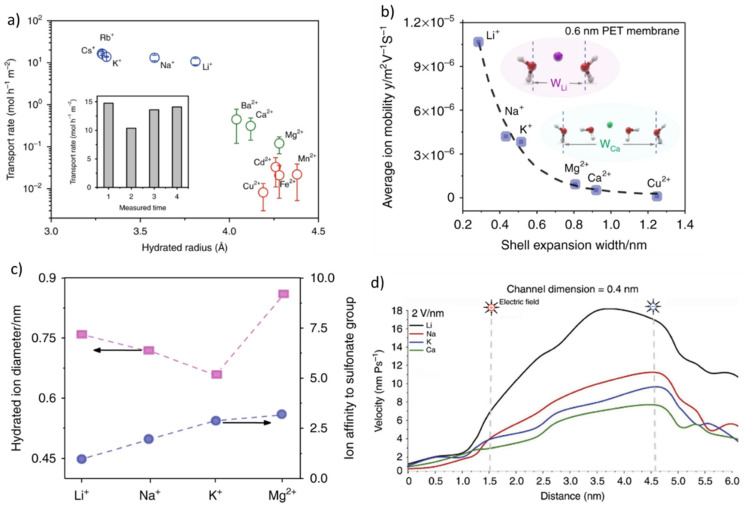
Effect of nanochannel size of Li-ion selectivity (**a**). The ion mobility vs. hydration shell (**b**). The hydration ions diameter of light metal cations (**c**). Comparison of velocity of Li^+^, Na^+^, K^+^, and Ca^2+^ in 0.4 nm vermiculite nanochannel (**d**) Reprinted based on the open access license from [72,75].

Ion selectivity across ion exchange membranes is directly related to the chemistry and morphology of the micropores within the material. An electric double layer (EDL) will be formed across the surface of the pores within ion exchange membranes, which is characterized by the Debye length, a variable depending on the ionic strength of the solution and the distance between the surface and charge species. The EDL, the depth of which may vary from ~1 nm to a few tens of nano-meters, consists of the Stern and Helmholtz layers, which correspond to either polarized or diffuse layers, respectively [72]. The electrical attraction generated by the diffuse layer is weaker than that of the polarized layer, which means that counter ions can diffuse with limited resistances. These interactions are typically measured in terms of the streaming or zeta potential, which decay exponentially concerning the inverse of the distance from the surface [72]. The ion selectivity within the pores may therefore be explained by accounting for differences between the hydration free energy of the ion and the energy of interaction between the ion and the charged site within the micropores [73,74]. The anionic field strength of the binding sites is the critical factor determining the selectivity sequence of the micropores for a series of cations. A typical selectivity sequence ranges from Li+ > Na+ > K+ > Rb+ > Cs+, while at the lowest anionic field strength, the micropores, corresponding to the free volume between the macromolecular chains of the ion exchange resins, may reverse the selectivity sequence as follows: Li+ < Na+ < K+ < Rb+ < Cs+ (Figure 3a).

The size of the free volume, the charge of the surface, and the external driving forces applied across the membrane stack will influence the rate of diffusion and the perm-selectivity of Li ions diffusion compared to other cations across membranes [72]. The dimension of the micropores and the loss of the hydration shells of the ions upon entering the channels are crucial to diffusion since these critical dimensions are typically smaller than the hydrated radii of most alkali metal ions (Figure 3b). The charge distribution and densities across the micro-channels will also dictate the rate of ion transfer and negatively charged moieties, and polymer backbones should be used for cation diffusion and to repel anions. The impact of the pendant cation exchange groups across ion exchange resins was evaluated to optimize Li+ ion perm-selectivity. Sulphonate [76,77], carboxylic [78], as well as hydroxide groups were found to offer weak interactions supporting ion hopping (Figure 3c). Ion affinity to -SO_3_^−^ was found to follow the trend Mg^2+^ > K^+^ > Na^+^ > Li^+^, thus promoting the facile release of Li ions.

The application of the external electric field during ED promotes the migration and depletion of ions from the feed side across the membrane stack. The enrichment of ions across the membranes and on the permeate side results in a strong polarization. This mechanism, which creates electro-convection at the membrane surface but also water stripping, may lead to changes in ion selectivity. Such limiting considerations in designing Li-ion-selective membranes are created due to the high diffusivity and response rate in Li to variations in current densities (Figure 3d) [72].

#### 3.1.2. Li extraction Case Studies with ED

Lithium extraction from brines by ED has been demonstrated from model solutions, industrial wastewaters, and natural lake waters. The impact of the Mg^2+^/Li^+^ ratio, feed temperature (15 to 30 °C), feed flow rate, solution residence time, and current densities across the membrane stack (5.9–13.8 A/m^2^) were systematically investigated. The speciation between Li^+^ and Mg^2+^ was achieved at high Mg^2+^/Li^+^ ratios. The Mg/Li mass ratio decreased as high as 21.8 times for the mixture with initial mass ratio of a Mg/Li of 400 [79]. In this research the commercial ion exchange membranes Asahi Glass Selemion CSO and ASA were applied. The influence of cations other than lithium ones affected the separation efficiency at different concentrations of Na^+^, Mg^2+^, and sulfates. The specific transfer mechanism of lithium could be related to the presence of sulfate ions. The mass transfer through the ion-exchange membrane of each ion species was determined by its dominant existing form [80].

The influence of the presence of coexisting species on the speciation of Li ions across cation exchange membranes was studied. Neosepta CIMS membranes were used for selective extraction of Li ions in mixed liquors containing other ions. The results showed sequences of coexisting cations, in the series K^+^ > Na^+^ > Ca^2+^ > Mg^2+^, directly affected separation behaviors of lithium. Interestingly, the higher the concentration of the mixed competing monovalent cations, the lower the selectivity for Li-ion was reported. The presence of sulfate and carbonate anions promoted Li over Mg fractionation. Furthermore, the presence of the coexisting anions affected the migration of Mg^2+^ [4].

The extraction of Li^+^ ions from lithium bromide solutions contaminated with Na+ ions was demonstrated for industrial liquors where lithium bromide is used as a working liquid within absorption chillers [81]. Although the feed solution contained ~13 g/L of Li ions and 1.35 g/L of Na ions, concentration factors of 88 were achieved for Li/Na. The ratio for fresh and unpolluted lithium bromide solution was 58 [81]. The separation of Mg^2+^ from Li^+^ ions was evaluated in terms of separation efficiency and economic benefit, with monovalent ion-exchange membranes. At an optimal applied ED cell voltage of 5 V and a pH range of 4–5, the Li-ions recovery reached 75.44% [82]. The modification of commercial membranes to improve lithium transport with ionic liquids (N,N,N-trimethyl-N-propylammonium–bis(trifluoromethanesulfonyl) imide (TMPA–TFSI) the Selemion CMV) was evaluated [83]. The application of selective cation exchange membranes was also evaluated. The electrodialysis voltage was 2–3 V, and the process was run for up to 15 h. After this time, 63% of the lithium was separated from the Li, Na, Mg, and K ions mixture [84].

Spent battery effluents were treated by ED to support Li-ions extraction [85]. The solution was first purified and lithium precipitated with phosphate to obtain Li_3_PO_4_. The selective separation of lithium over phosphor was achieved [85]. Li-ion recovery from spent battery effluents containing Co ions was performed with multi-stage metal-ion chelation and the ED process. Ethylenediaminetetraacetic acid (EDTA) was added to cause the selective chelation of Co ions and to increase the concentration of Li ions in the permeate stream [86]. Lithium and cobalt separations with monovalent selective ion exchange membranes such as PC-MVK were demonstrated. The value of the applied potential did not influence significantly the separation efficiency: the rise in voltage from 5 V to 15 V turned the separation factor from 98.6 to 99.4% [87]. The cobalt ion concentration in the feed solution affected the selectivity of the monovalent ion exchange membrane. Some reports on the use of electrodialysis for lithium recovery are summarized in Table 7.

### 3.2. Capacitive Deionization (CDI)

Capacitive Deionization is an electro-adsorption technique developed in the late 1970s for the removal of ions from aqueous solutions by electrosorption on porous material [90,91]. CDI has been primarily applied to seawater and brackish water desalination, sewage remediation, as well as in the softening of drinking water [92,93].

#### 3.2.1. Operation of CDI Systems

**Figure 4:**
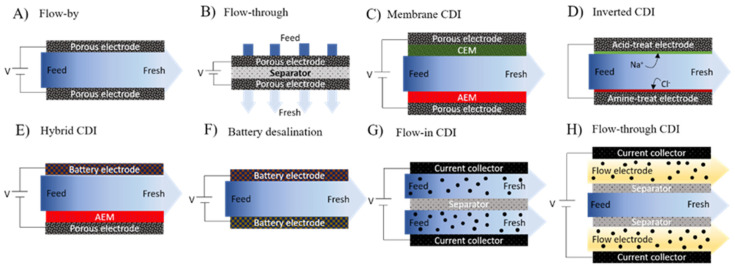
Cell composition of CDI family cells.

Electro-active adsorbent materials, such as those used in CDI, mostly involve physisorption at the solid–liquid interface to support ultra-selective extraction of resources [90,92,94,95,96]. Typical CDI configurations are shown in Figure 4.

**Figure 5:**
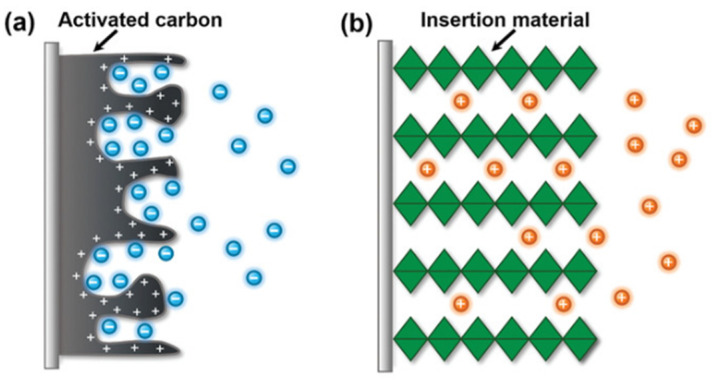
Two type of ions accumulation in (**a**) activated carbon according with EDLs mechanism and (**b**) intercalation and de-intercalation ions according with Faradaic reactions. Reprinted based on the open access license from [95].

The most popular material for CDI electrodes is activated carbon (AC) due to its low cost, high electrical conductivity, and large specific surface area. However, AC does not exhibit any selectivity toward ions due to the absence of selective sites (Figure 5a). To generate Li+ ion selectivity, the surface of activated carbon could be modified by selective moieties such as α-MnO_2_ [96].

The ion insertion reaction across AC is due to the diffusion into interstitial sites of the electrode material through a Faradaic charge-transfer process (Figure 5b). The MnO_2_ materials can store lithium ions via two types of electrochemical processes, namely, surface-dependent Faradaic reaction, or pseudo-capacitance, and insertion into the bulk material. Highly crystallized MnO_2_ materials have 1D-, 2D-, or 3D-type tunnels built from MnO_6_ octa-hedric assemblies that support the intercalation. Their spacing may be controlled by doping such metal as Ti or Fe [97,98]. Electrochemical lithium recovery was introduced to extract lithium from Li^+^ ions from geothermal or industrial brines. The method requires lithium-selective materials to recover lithium, such as LiFePO_4_/FePO_4_, MnO_2_, and lithium manganese oxides [99].

The use of nickel hexacyanoferrate (KNiFe(CN)_6_) as a Li^+^ exclusion electrode material [100] was considered since nickel hexacyanoferrate has a higher affinity toward such ions as Na^+^ or K^+^ rather than for Li^+^. With this strategy, seawater can be used as a recovery solution and reduces the consumption of freshwater. By optimizing the CDI process variables, lithium enriched streams were obtained, and a lithium recovery of 73% was obtained. The process was characterized by an extremely high salt adsorption capacity of 800 mg/g and total energy consumption of 0.183 W h/g of adsorbed salt [7]. The development of lithium iron manganese oxide electrodes as selective materials to facilitate Li^+^ release [101] was evaluated, and it obtained an over 70% Li recovery. The ratio Na:K:Li changed from 227:1.1:1 in feed to 2.9:0:1 after one cycle of separation [101].

#### 3.2.2. Performance/Materials Relationships for Li Recovery in CDI Systems

In a classic CDI system, the cell is composed of a pair of porous membrane electrodes sandwiched between a separator that facilitates the flow of liquids and prevents electrode physical contact of electrodes [102,103]. A typical CDI system is composed of a porous carbon electrode and a current collector. The surface of the CDI electrode may be further functionalized with specific chemical moieties or polymers to enhance ion selectivity [83,84]. The addition of ion-exchange species on porous electrodes enables selective ion capturing and prevents re-adsorption during the discharge of the electrode [80]. The nature of the functional groups located on the CDI scaffold affects the electrostatic interactions with ions [85,86], reduces energy requirements [87], and improves the process stability [85].

Coating of carbon electrodes with LiMn_2_O_4_ was performed to support Li recovery from lithium hydroxide solutions. The desorbed lithium ions from the modified MCDI system were found to be 8.7 mg/g at a constant voltage of 3.5 V, and this was lower (by approximately 45%) than the desorption for the conventional process with acidic solution [104]. A cathode composed only of LiMn_2_O_4_ was also developed [105]. The maximum salt adsorption capacity (SAC) was estimated at 24 mg of lithium per 1 g of electrode material. Moreover, this process did not require the use of the acidic solution in the desorption process [105].

Selective lithium recovery from multi-component aqueous solutions (Li^+^, Na^+^, K^+^, Ca^2+^, and Mg^2+^) reached 0.22 μg/g (with applied 1 V constant voltage electric mode) and was 7 times higher than that from a control physio-sorption process running (without application external electrical field) in similar experimental conditions during the CDI process. During application 1 V, the recovery amount of Li^+^ reached 350 µmol/g_adsorbent_. When the electrical field was not applied, the recovery reached only 50 µmol/g_adsorbent_. The energy required for the recovery was estimated to be 23.3 W h/g of lithium. Moreover, manganese dissolution was not observed during five consecutive recovery cycles supporting the scalability and reproducibility of the process [106]. Monovalent selective cation exchange membranes (Neosepta CIMS, Astom Corporation, Japan) were tested for various rates of lithium over magnesium ions in feed solutions. The maximum performance was found to be 38.4% of recovered Li with an energy consumption 0.36 W h/g of lithium [107].

A hybrid capacitive deionization (HCDI) process has been performed with lithium titanium manganese oxide as the cathode material. The anode material was modified by adding polypyrrole (PPy) to increase the conductivity of the material. An electro-sorption capacity for LiCl of 36.9 mg/g was achieved while the corresponding capacities for NaCl and KCl were 18.09 mg/g and 9.07 mg/g, respectively [108]. Modified anion exchange membrane (AEM) was produced by chemical grafting of poly(vinyl chloride) (PVC) with aliphatic amines. The extraction of 40 mg/g of LiCl was obtained in comparison to 10 mg/g for NaCl. A recovery of about 50% of lithium was noted [94,109]. Poly(vinylidene fluoride) materials were evaluated as a supporting polymer for the AEM preparation. The salt adsorption capacity of the optimized materials was estimated at 30 mg/g with 0.9 current efficiencies and 96% of desorption efficiency [110].

The active cathode material is the next important element of the CDI system. Lithium–manganese–titanium oxide (LMTO) with varying concentrations of titanium dioxide (TiO_2_) has been tested as a cathode [98]. The best-performing material, which contained 5% of TiO_2_, had a sorption capacity of 36 mg/g, and the uptakes of KCl and NaCl were 16 and 11 mg/g, respectively. Additionally, this adsorbent needed two times less energy for the recovery of lithium chloride than other monovalent salts [98]. That adsorbent was used for selective recovery of Li from geothermal waters of the Western Carpathian Mountains region [7]. Lithium recovery of 73% was achieved with an extremely high salt adsorption capacity of 800 mg/g and total energy consumption of 0.183 W h/g [7]. Lithium–iron–manganese adsorbents, with varied ratios of Li/Mn and Li/Fe, were tested for a similar feed [101]. The adsorbents with the molar ratios of Li/Mn and Li/Fe of 1.5:1 showed the best salt adsorption capacity for LiCl. Moreover, 32 mg/g of lithium, 16 mg/g of sodium, and 0 mg/g of potassium was found. The use of a modified electrical protocol, with double stage of desorption, as found to be a good method for lithium recovery from solution. The recovery reached the efficiency of 76% and reduced the Na:K:Li-ions ratio from 227:1.1:1 at the feed to 2.9:0:1.

The CDI process with flowing electrodes (FCDI) was investigated with adsorbing materials developed from reduced graphene oxide and mixed metal oxides and activated carbon. The suspension of fine particles of reduced graphene oxide formed the cathode, and the suspension of the activated carbon formed the anode [106]. A lithium extraction efficiency of 13.684 mg/g was obtained, and the energy consumption per lithium was as small as 0.22 W h/g of Li. The overall process led to 93% of lithium-ion recovery from the model brines, which indicated that the investigated materials could be promising in the recovery of lithium from natural and battery leachate solutions.

A summary of CDI processes for lithium recovery is presented in Table 8.

### 3.3. Hybrid Membrane Systems Involving Electro-Membrane Processes

Greater lithium-ion recoveries from any feed source may be achieved through a combination of processes into the treatment trains. Such approaches may not only support a more cost-effective Li extraction but also support higher product purity, lower energy consumption, and safer operation resulting in the more sustainable technologies. The comparison of described methods is presented in Table 9.

#### 3.3.1. Electrodialysis (ED)–Reverse Osmosis (RO)

Integration of reverse osmosis and electrodialysis was used for lithium recovery from wastewater [113]. The RO concentrate was used as feed for the ED process. It was noted energy reduction from 26.67 to 7.81 kW h/m^3^ was achieved. An obtained enrichment concentration factor of 12.32 showed the feasibility for the use of this approach to produce high-volume concentrate.

#### 3.3.2. Ion Exchange Adsorption–Ultrafiltration (UF)

A process coupling ion-exchange adsorption and UF was developed to support Li recovery from geothermal waters. λ-MnO_2_ was produced from spinel-type lithium manganese dioxide, grounded to fine particles, and used in a concentration of 1.5 g adsorbent/L. The authors identified advantages of the use of ion exchange–UF hybrid for the separation process of lithium from geothermal water [20,114].

#### 3.3.3. Adsorptive Ion Exchange Membranes

Another approach for selective Li-ion extraction is to combine mass transfer through ion exchange membranes and adsorption within an adsorptive lithium-selective membrane [115]. This type of material enables the separation of Li ions from brines at enrichments concentration factors up to 62,000 compared to less than 100 for other metal ions. This type of membrane adsorbent may concentrate Li-ions efficiently from seawater even though the native Li-ions concentration in such effluents is very small in comparison to Na^+^, K^+^, Mg^2+^, or Ca^2+^ ions. Most of the metal ions adsorbed on the membrane were desorbed to the solution by the treatment with a 0.75 M HCl solution. The desorbed fractions contained 95%, 95%, 93%, and 93% of Na^+^, K^+^, Mg^2+^, and Ca^2+^ ions, respectively [115].

#### 3.3.4. Membrane Distillation Crystallization

The process employed a membrane distillation (MD) and crystallization process is called membrane distillation crystallization (MDC). Compared with the traditional crystallization process, the MDC displays rapid crystallization and well-controlled nucleation kinetics. The MDC was investigated to recover salt crystals from a single-salt LiCl solution. The required concentration of precipitate the LiCl should be over 14 M. The MDC reached only 10 M. The required concentration level is possible by applying the vacuum membrane distillation [116].

#### 3.3.5. Leaching–Flotation–Precipitation Process

The stepwise leaching–flotation–precipitation process was adopted to separate the Li/Fe/Mn from batteries [117]. First, the cathode material was leached according to the acid leaching procedure. Then, the Fe^3+^ cations are selectively floated and recovered as a FeCl_3_ in the flotation step. Finally, the Mn^2+^/Mn^3+^ and Li^+^ cations are precipitated and separated as MnO_2_/Mn_2_O_3_ and Li_3_PO_4_ using saturated KMnO_4_ solution and Na_3_PO_4_, respectively. As a result, the total recovery of Li, Fe, and Mn is ~81%, ~85%, and ~81%, respectively. Hence, that stepwise process could be considered an alternative way to separate and recover metals from spent Li-ion batteries effectively.

#### 3.3.6. Membrane Electrolysis

The membrane electrolysis was investigated to crystallize lithium carbonate from lithium-rich brines. The three-compartment reactor was applied. The brines were introduced in the middle compartment, separated from the anolyte and catholyte compartment outside. When a current is applied, anions and cations selectively migrate into the anionic and cathodic compartments, respectively. Water reduction increases the pH of the catholyte, which is recirculated in a crystallizer where CO_2_ is bubbled and converted to carbonate, precipitating Li_2_CO_3_ with a purity of at least 93.8 wt%. The method allows recovering as much as 90% of the lithium-containing solution volume as low salinity water, with up to 99.7% less total dissolved solids than the processed brine, in marked contrast with current practice [118].

#### 3.3.7. Membrane with Incorporated Metal–Organic Frameworks (MOF-on-MOF)

The distinguished research was conducted on adapting the biological ion channels features to the alkali metal ions recovery (Na^+^, K^+^, and Li^+^). The main concept was the fabrication of monovalent ion-selective membranes with asymmetrical sub-nanometer pores dedicated to transportation lithium cations. The ionic current measurements exhibit an unprecedented ionic current rectification ratio of above 100 with exceptionally high selectivity ratios of 84 and 80 for K^+^/Li^+^ and Na^+^/ Li^+^, respectively (1.14 Li^+^ mol m^−2^ h^−1^) [119].

#### 3.3.8. Pervaporation

The modified by incorporating graphene oxide (GO) into polypropylene hollow fiber membranes (Accurel PP S6/2, from Membrana GmbH, Germany). With a high initial feed concentration (>200 g/L of salt) the GO composite pervaporation membrane increased lithium concentration from 0.3 to 1.27 g/L (73% feed volume reduction) [120].

## 4. Economical Aspects of Lithium Recovery with Electro-Driven Membrane Processes

Techno-economic analysis of lithium production based on three main sources of lithium: Namely, minerals, brines, and e-waste, is also discussed in the following sections. Relationships between the operating conditions and the required performance are developed to shed light on the energy requirements for each source of lithium ions and the results for traditional hydrometallurgical technologies and electro-driven membrane processes or hybrid solutions are compared.

### 4.1. Lithium Recovery from Minerals

**Figure 6:**
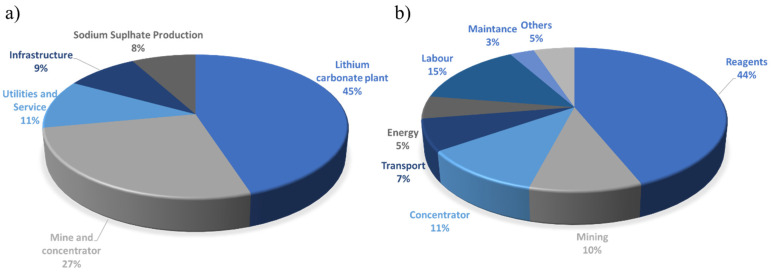
The distribution of shares of the individual components for lithium recovery from minerals (**a**) and brines (**b**) [121].

Traditionally, for the extraction of lithium salt to produce lithium carbonate from minerals the leaching acid, alkaline, chlorination, and a combination of these techniques are applied. The cost factors include mine and concentrator development and construction. The distribution of cost among the individual components is shown in Figure 6a.

The pie chart shows that 45% of lithium recovery costs are related to the lithium carbonate plate cost. The second most expensive component is the mine and concentrator. The dominating costs within the lithium carbonate plant are reagents, labor, and energy costs [121].

### 4.2. Lithium Recovery from Brines

For lithium recovery from brines, the evaporation methods were chosen as the main technology at the industrial scale. Within this technique, the evaporation ponds costs play an important role. The second place takes lithium carbonate plate with its utilities and infrastructure (Figure 6b) [121].

From the operational cost, the reagents’ costs seem to be the most important part. Among them, sodium carbonate (28%), calcium oxide (12%), sodium hydroxide (7%), carbon dioxide (4%), and hydrochloric acid (1%) should be mentioned [121].

### 4.3. Lithium Recovery from e-Waste Brines

**Figure 7:**
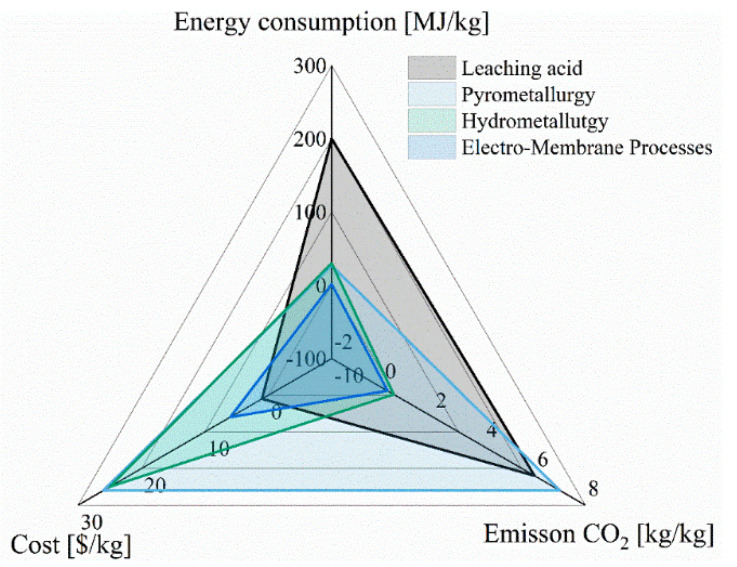
Economic analysis for methods of recycling of LIBs.

The lower cost of process utilization battery spent solutions is expected to be the main driver for recycling end-of-life LIBs. From a typical economic analysis of LIB recycling delivery, the total cost of recycling 3974 tons of LIBs was USD 22,824,666. The operation cost was USD 8,941,500 (2250 USD/ton), transportation was USD12,078,970 (USD 3039.5/ton), and material handling was USD 1,804,196 (454 USD/ton) [34]. Environmental and social aspects could also contribute to the need for more recycling of end-of-life batteries. The key areas for the reduction in cost are related to energy and greenhouse gas emissions. Figure 7 summarizes some values from several studies comparing processes involving leaching acid, pyrometallurgy, hydrometallurgy, and electro-membrane processes [122,123,124].

Based on the data from these studies, the most efficient method, with the lowest carbon footprint, is offered by the electro-membrane processes. On the other hand, the most expensive technologies for lithium recovery are pyrometallurgy and hydrometallurgy. This is due to the high energy requirements for heating and extraction.

The comparison of all of the above-mentioned methods is difficult due to the usability of various operations, materials, and energy requirements.

## 5. Summary and Prospects

Lithium’s unique properties make it a critical metal for a wide range of applications. The demand for Li compounds in the commodity market over the next decade and beyond is expected to increase dramatically according to the rising use of portable energy storage devices. At this stage, there are already some industrial-scale or laboratory-established technologies for recovering lithium from minerals, brines, and lithium-ion batteries. The process of lithium recovery from minerals and clays is expensive at the both mining costs and energy consumption. The main source of Li from minerals is spodumene. Spodumene has a high energy requirement to convert α-spodumene into β-spodumene, which is more readily leachable. The extraction of lithium from brines and seawater reveals that a very long duration is necessary for evaporation and concentration. That process has a serious drawback and is seriously affected by climate. The recycling process of the LIBs mainly consists of dismantling for the removal of plastic and iron scraps, the separation of cathode and anode materials, the leaching of the electrode, the removal of unwanted metal impurities in the leachate, separation, and the recovery of metals from the solutions by solvent extraction, ion-exchange, and precipitation. Electro-membrane processes could be applied for lithium removal from brines and spent batteries. For brines and groundwater, capacitive deionization can be efficiently applied. By using CDI and HCDI, it is possible to reduce energy consumption, as well as intensify removal operations with high selectivity. For releasing lithium from e-wastes, the ED process can be used. By applying ED, it is possible to reduce recycling costs and energy consumption. The additional benefit of ED over other technologies is the extraction of is the extraction of lithium in higher-grades. However, despite the promise of electro-membrane processes for lithium recovery, there is a need to continue research on the development of sustainable technologies that can effectively recover all valuable metals from both primary and secondary resources, simplify the recycling process, and make the recycling costs lower.

## Figures and Tables

**Table 1:** Chemical composition with the percentage of lithium in minerals [16,17].

Minerals	Chemical Formula	Percentage of Lithium (wt%)
Spodumene	LiAlS_2_O_6_	3.73
Petalite	LiAlSi_4_O_10_	2.27
Lepidolite	LiKAl_2_F_2_Si_3_O_9_	3.56
Amblygonite	LiAlFPO_4_	4.74
Eucryptite	LiAlSiO_4_	5.53
Abnormalite	LiCO_3_	18.75

**Table 2:** The most common lithium salt pools [17,18].

Country	Reservoir	Li Content (wt%)
Chile	Atacama	0.15
China	Zabuye	0.097
Chile	Maricunga	0.092
Argentina	Olaroz	0.07
Argentina	Hombre Muerto	0.062
Bolivia	Uyuni	0.045
USA	Great Salt Lake	0.04
USA	Smackover	0.037
China	DXC	0.033
USA	Silver Peak	0.03
Global	Geothermal water *	0.015
Canada	Fox Creek	0.01
Israel	Dead sea	0.002
Global	Sea *	0.00017

* The average concentration.

**Table 3:** The weight percentage of Li in each part of LIB material [22].

Component	g	%
Cathode material	130.9	41.4
Metallic shell	51	16.1
Plastic shell	50	15.8
Electrolyte	20.9	6.6
Cu electrode	17.2	5.4
Al electrode	7.5	2.4
Polymer	6.8	2.2
Total	316	100

**Table 4:** Comparison of leaching processes for lithium extraction from minerals [5,36,43].

Process	Acid/Sulfonation	Alkali	Chlorination
Active reagents	Alkali metal sulphates, sulfuric acid, SO_3_ at water or oxygen	Lime or limestone	Hydrochloric acid, sodium chloride, calcium chloride, or chlorine gas
Time	1–3 h	1–2 h	Up to 2.5 h
pH	2–3	8–10	~5
Temperature	200–1000 °C	100–200 °C 800–1000 °C	800–1100 °C
Disadvantages	Non-selective method; A lot of leached solution is needed; Impurities such as Al, Na, Mg, Fe, and K	Need to decompose lime and limestones to CaO	Toxic chloric reagents; aggressive environment of leaching
Advantages	High rate of Li extraction	High rate of Li extraction without corrosion agents	Selective for lithium chloride production

**Table 6:** Comparison of different processes for recycling spent LIBs [67].

Process	Operations	Advantages	Disadvantages	Company
Pyrometallurgy	Mechanical crushing Thermal processing including calcination process, roasting process, reduction process, and chlorine process	Easy to scale up; simple pre-treatment; acid/alkaline free	High energy consumption; emission toxic gases and dust; hard to achieve lithium recovery	Accure GmBH; Batrec Industrie AG, Umicore; Inmetco, Akkuser Ltd.; SNAM
Hydrometallurgy	Discharge and dismantling Leaching processes including chemical precipitation. solvent extraction, sol–gel reactions, electrochemical processes	Easy to recycle lithium; less gas and dust emissions; high purity of products	Consumption of acid/alkaline; low efficiency; complex to purification/separation metals	Retriev/Toxco; Recupyl; AEA; Onto
Hybrid processes (Direct processes)	Mechanical process Mixed processes including pyrometallurgy, hydrometallurgy, and pyrometallurgy	Relatively low efficiency of energy consumption; satisfactory recycling efficacy	Emission of toxic gasses and dust; complex process operations	Sony/Sumitomo
Bio-metallurgy	Pre-treatment processes Bio-leaching including chemical precipitation, solvent extraction, and electrochemical processes	Low cost; environmentally friendly	Time-consuming; possibility of contamination; sensitivity of microorganisms for pH and temperature	Have potential for commercialization

**Table 7:** Electrodialysis process for lithium separation from aqueous solutions.

Method	Electrical Mode	Lithium Resources	Membrane Type	Perm-Selectivity	Li Ions Extraction Efficiency	Energy Efficiency	Advantage	Limitation	Ref.
Electrodialysis for brines	CC = 5.9 A/m^2^	C_Li_^+^ = 0.15 g/L C_Mg_^2+^ = 22.5 g/L Mg^2+^/Li^+^	Selemion CSO Selemion ASA	S_Li/Mg_ = 20.2–33.0	>90%	1.9 Wh/g_Li_^+^	High selectivity for lithium ions, eco-friendly		[79]
	CV = 6.0 V	C_Li_^+^ = 0.15 g/L C_Mg_^2+^ = 22.5 g/L Mg^2+^/Li^+^ = 150	Selemion CSO Selemion ASA	S_Li/Mg_ = 17.9	96.1	0.78 Wh/g_Li_^+^			[80]
	CV = 12–28 V	C_Li_^+^ = 4.5 g/L C_Mg_^2+^ = 85 g/L Mg^2+^/Li^+^ = 18.9	Selemion CSO Selemion ASA	S_Li/Mg_ = 9.89	90.5	4.5 Wh/g_Li_^+^	Application model real brines from East-Taijiner	Non-equal mass balance	[80]
	CV = 5 V	C_Li_^+^ = 1 g/L C_Na_^+^ = 1–30 g/L C_Ca_^2+^ = 1–30 g/L C_Mg_^2+^ = 1–30 g/L	Neosepta CIMS Neosepta ACS	S_Li/Mg_ = 20	80	4.7 Wh/g_Li_^+^	Microcosmic theory of separation lithium	Different perm-selectivity depends on the initial ratio lithium to other cations	[8]
	CV = 3–8 V	Mg^2+^/Li^+^ = 20	n.s.	S_Li/Mg_ = 3.5–4.2	60	62 Wh/g_Li_^+^	Effect of coexisting cations on lithium separation	High energy consumption	[4]
	CV =7 V	C_Li_^+^ = 12–15 g/L	AR204SXR412 and CR67, MK111 (Ionics, MA, USA)	n.s.	20	n.s.	Separation lithium toward sodium ions	Low efficiency of lithium recovery	[81]
	CV =5 V	C_Li_^+^ = 0.14 g/L C_Na_^+^ = 8.4 g/L C_Mg_^2+^ = 3.04 g/L C_Cl-_ = 30.25 g/L	Neosepta CIMS Neosepta ACS	S_Li/Mg_ = 13	75.44	28.16 Wh/g_Li_^+^	Separation lithium and Magnesium	High ratio of Magnesium in product	[82]
	CV = 2–3 V	C_Li_^+^ = 0.17 µg/L C_Na_^+^ = 105 µg/L C_Mg_^2+^ = 13.5 µg/L C_K_^+^ = 3.8 µg/L	Selemion CSO Selemion CMV	n.s.	63	n.s.	Separation lithium from seawater	Low ratio of recovery	[83]
	CV = 2 V	n.s.	Li ionic superconductor-type crystals such as Li_1+x+y_Alx (Ti, Ge)_2−x_SiyP_3−y_O_12_, (Li_x_, La_y_) TiO_z_ and (Li_x_, La_y_) ZrO_z_ can be used as LISMs	n.s.	7	n.s.	Separation lithium from model mixture of Na, ka, Mg, and Ca	Low ratio of recovery	[84]
Electrodialysis with bipolar membrane	CV = 15 V	C_Li_^+^ = 250 mg/L C_boron_ = 800 mg/L	Standard CEM PC SK, bipolar membrane PCCell bipolar type PC bp and AEM PC Acid 60	n.s.	99.6	n.s.	Separation of boron and lithium from aqueous solution	Not specified energy consumption	[19]
	CV = 30 V	C_Li_^+^ = 340 mg/L C_boron_ = 1000 mg/L	Neosepta BP-1E Neosepra CMB Neosepta AHA	n.s.	94.7	7.9 kWh/m^3^	Separation of boron and lithium from aqueous solution	Higher energy consumption than in classic ED	[88]
	CV = 6 V		Neospeta CMX Neosepta BP-1	n.s.	60	n.s.	Recovery lithium from lithium manganese oxide by BMED	Multistage processes with pre-treatment and desorption	[69]
	CC = 20–60 mA/cm^2^	n.s.	JAM-II-05 JCM-II-05 Neosepta CMX Neosepta BP-1	n.s.	n.s.	n.s.	Application Electro-electrodialysis bipolar membrane for production lithium carbonate		[89]
Electrodialysis for LIBs		C_Li_ = 3.27 g/L C_Al_ = 0.23 g/L C_Co_ = 0.46 g/L C_Cu_ = 0.68 g/L C_Mn_ = 0.28 g/L C_Ni_ = 0.25 g/L C_Zn_ = 0.78 g/L C_Cl_ = 17.5 g/L	DuPont Nafion-117	n.s.	90	27 Wh/g_Li_^+^	Application ED for lithium battery spent utilization	Mulistage process with purification, precipitation, dissolution, electrodialysis and ion exchange reaction	[85]
		C_Li_ = 1.3 g/L C_Co_ = 1.2 g/L C_EDTA_ = 9 g/L	Selemion CMV Selemion AMV Neosepta BP-1E	n.s.	99	n.s.	High ratio of separation Li and Co	Application a chelating agent	[86]
	CV = 5 V	C_Li_ = 0.1 g/L C_Co_ = 0.3 g/L	PC-MVK PC-MVA	n.s.	99.4	n.s.		Scaling of IEMs	[87]

**Table 8:** Comparison of CDI techniques for lithium removal from brines.

CDI Configuration	Sources of Li^+^	Selective Element of CDI Cell	Electrical Mode	Concentration of Feed Composition [mg/L]	SAC [mg/g]	Energy Consumption [Wh/g_Li_^+^]	Ref.
MCDI	Brine model solution without acid	Membrane with lithium adsorbent incorporation	CV = 3.5 V	C_LiOH_ = 60	8.7	n.s.	[104]
MCDI	Brine model solution without acid	Modified cathode with LiMn_2_O_4_	CV = 1.0 V	C_LiOH_ = 50	24	n.s.	[105]
MCDI	Simulated Atacama brine	Modified cathode with LiMn_2_O_4_	CV=1.0 V	C_Li_^+^ = 1.35 C_Na_^+^ = 7590 C_K_^+^ = 17.9 C_Mg_^2+^ = 9.6 C_Ca_^2+^ = 1.6	0.0022	23.3	[106]
MCDI	Brine model solution without acid	Monovalent selective membrane, CIMS Neosepta	CV = 0.6–1.4 V	C_Li_^+^ = 37	n.s.	0.36	[107]
HCDI	Brine model solution without acid	Modified cathode by lithium titanium manganese oxide	CV = 0.7 V	C_Li_^+^ = 63.9	33.4	n.s.	[108]
HCDI	Brine model solution without acid	Modified cathode by lithium titanium manganese oxide	CV = 2.5 V	C_Li_^+^ = 63.9	40	n.s.	[94,109]
HCDI	Brine model solution without acid	Modified cathode by lithium titanium manganese oxide	CC = 10 A/m^2^	C_Li_^+^ = 63.9	30–40	n.s.	[110]
HCDI	Brine model solution without acid	Modified cathode by lithium titanium manganese oxide with different ratio of titanium oxide	CV = 1 V	C_Li_^+^ = 63.9	36	120 Wh/m^3^	[98,111]
HCDI	Real geothermal multicomponent solution	Modified cathode by lithium titanium manganese oxide with 5% of titanium dioxide	CV = 2 V	C_Li_^+^ = 15.7 C_Na_^+^ = 10,298 C_K_^+^ = 102.1 C_Mg_^2+^ = 50.3 C_Ca_^2+^ = 63.7 C_Sr_^2+^ = 33.5	800 (total)	0.183 Wh/g	[7]
HCDI	Model geothermal multicomponent solution	Modified cathode by lithium iron manganese oxide with different ratio of Li/Mn, and Li/Fe	CC = 0.7 A/m^2^	C_Li_^+^ = 25.9 C_Na_^+^ = 5895 C_K_^+^ = 29.6 C_Mg_^2+^ = 24 C_Ca_^2+^ = 16	318 (total)	n.s.	[101]
FCDI		rGO/LiNi_0.6_Co_0.2_Mn_0.2_O_2_	CV = 3.3–4.5 V	C_Li_^+^ = 3.67 C_Na_^+^ = 11 C_K_^+^ = 1.2 C_Mg_^2+^ = 5 C_Ca_^2+^ = 0.015	13.84	0.22 Wh/g_LI_^+^	[112]

**Table 9:** Comparison of the hybrid process of lithium extraction.

Hybrid Processes	Advantages	Disadvantages
Electrodialysis–Reverse osmosis (ED-RO)	○ High ratio of removal ○ Selective ○ Continuous operation	○ High energy consumption ○ High pressure is required (RO)
Ion exchange adsorption–ultrafiltration	○ Selective	○ Deterioration of sorbent (MnO_2_) ○ Limited sorption ○ Expensive
Adsorptive ion exchange membrane	○ High selective ○ Continuous operation	○ Required desorption step ○ Deterioration of active material
Membrane distillation crystallization	○ Cost-effective ○ A market valuable form of Li salts is produced	○ A high concentration (14 M) is required for precipitation
Leaching–flotation–precipitation process	○ High separation and selectivity	○ The number of disposals is high ○ Required aggressive environment
Membrane electrolysis	○ Continuous operation ○ High purity and separation	○ Complicated roces ○ Additional reagents are needed
MOF-based membrane	○ High selectivity ○ Continous operation	○ Difficulties in preparation
Pervaporation	○ With a ratio of lithium concentration ○ Cost-effective process ○ Easy to scale-up	○ Risk of fouling and scaling

## Data Availability

Not applicable.
